# A lightweight shape-memory alloy with superior temperature-fluctuation resistance

**DOI:** 10.1038/s41586-024-08583-7

**Published:** 2025-02-26

**Authors:** Yuxin Song, Sheng Xu, Shunsuke Sato, Inho Lee, Xiao Xu, Toshihiro Omori, Makoto Nagasako, Takuro Kawasaki, Ryoji Kiyanagi, Stefanus Harjo, Wu Gong, Tomáš Grabec, Pavla Stoklasová, Ryosuke Kainuma

**Affiliations:** 1 Department of Materials Science, Graduate School of Engineering, Tohoku University, Sendai, Japan; 2 Frontier Research Institute for Interdisciplinary Sciences, Tohoku University, Sendai, Japan; 3 Institute for Materials Research, Tohoku University, Sendai, Japan; 4 J-PARC Center, Japan Atomic Energy Agency, Tokai, Japan; 5 Institute of Thermomechanics, Czech Academy of Sciences, Prague, Czech Republic

**Keywords:** Metals and alloys, Mechanical properties

## Abstract

In advanced applications such as aerospace and space exploration, materials must balance lightness, functionality and extreme thermal fluctuation resistance^1,2^. Shape-memory alloys show promise with strength, toughness and substantial strain recovery due to superelasticity, but maintaining low mass and effective operation at cryogenic temperatures is challenging^3–6^. We hereby introduce a new shape-memory alloy that adheres to these stringent criteria. Predominantly composed of Ti and Al with a chemical composition of Ti_75.25_Al_20_Cr_4.75_, this alloy is characterized by a low density (4.36 × 10^3^ kg m^−^^3^) and a high specific strength (185 × 10^3^ Pa m^3^ per kg) at room temperature, while showing excellent superelasticity. The superelasticity, owing to a reversible stress-induced phase transformation from an ordered body-centred cubic parent phase to an ordered orthorhombic martensite, allows for a recoverable strain exceeding 7%. This functionality persists across a broad range of temperatures, from deep cryogenic 4.2 K to above room temperature, arising from an unconventional temperature dependence of transformation stresses. Below a certain threshold during cooling, the critical transformation stress inversely correlates with temperature. We interpret this behaviour from the perspective of a temperature-dependent anomalous lattice instability of the parent phase. This alloy holds potential in everyday appliances requiring flexible strain accommodation, as well as components designed for extreme environmental conditions such as deep space and liquefied gases.

## Abstract (web summary)

A Ti–Al-based shape-memory alloy adhering to the stringent criteria of lightness, functionality and extreme thermal fluctuation resistance is introduced, showing excellent superelasticity with a recoverable strain exceeding 7% and an ultra-wide temperature window from 4.2 K to 400 K.

## Main

Metallic materials capable of significant elastic deformation, without interference from plasticity or fracture, are not only vital for practical applications requiring strain accommodation^7,8^, but are expected to pave the way for strain-related physical or chemical property modification through elastic strain engineering^9^. However, most metallic materials can endure only small elastic strains of less than 0.5%. Shape-memory alloys, such as Ni–Ti, typically show large recoverable strains of around 10% owing to reversible phase transformations, known as superelasticity^3^. Certain alloy systems, such as Cu–Al–Ni, can demonstrate even larger recoverable strains, exceeding 17%, also due to superelasticity^10^. Among the many shape-memory alloys, Ti-based alloys are promising for various applications, offering a compelling combination of affordability, corrosion resistance and mechanical flexibility, particularly in medical fields^11^. Furthermore, the growing interest in space exploration by private companies and government agencies^12,13^, as well as the storage of liquefied gases such as liquefied hydrogen for future carbon neutrality^14^, creates an urgent demand for these lightweight structural and functional materials operable under extreme conditions such as cryogenic temperatures.

Beta-type Ti-based shape-memory alloys with body-centred cubic (BCC) crystal structures are particularly versatile within the Ti family. Ti-based shape-memory alloys have typically been developed by adding substantial amounts of denser elements such as Nb or Zr to stabilize the beta phase at room temperature^15,16^. However, this approach compromises the lightweight nature of Ti-based alloys. In addition, their recoverable strain in superelasticity is typically less than 3% (ref. ^15^), which is significantly smaller than that of commercial Ni–Ti shape-memory alloys of around 8% (ref. ^3^). Furthermore, the superelasticity of these alloys under extreme conditions, including cryogenic temperatures, is suboptimal^15^. A promising strategy for overcoming these limitations is the incorporation of Al, an even lighter element, into Ti to achieve a balance between lightness and enhanced mechanical properties. Alloys such as the widely used Ti–6Al–4V (mass %, Ti-64), which is a typical representative of the Ti–Al family, have been primarily developed as lightweight structural materials and sometimes as high-temperature materials^17^. However, they have not demonstrated superelastic properties with the absence of single beta phase at room temperature as one possible cause^17^. We have taken a closer examination of the Ti–Al binary phase diagram and have noticed a composition region with an ordered BCC (B2) phase at elevated temperatures^18^, a characteristic commonly associated with many shape-memory alloys^3,19,20^. By following thermodynamic guidelines based on the phase diagrams, incorporating less than 5 at% of Cr into the Ti–Al matrix and stabilizing the beta phase at room temperature through rapid quenching from high temperatures^21,22^, we have successfully synthesized a new Ti–Al-based shape-memory alloy with robust properties for temperature change.

## Superelasticity at room temperature

**Fig. 1:**
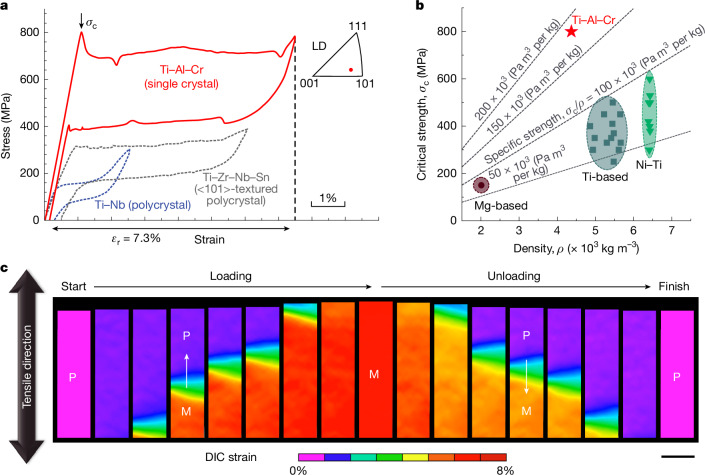
Superelastic properties of a near-<110> single-crystal Ti–Al–Cr alloy. **a**, Tensile loading–unloading engineering stress–strain curves of Ti–Al–Cr, Ti–Nb and Ti–Zr–Nb–Sn alloys at room temperature^15,16^. The upper inset shows the orientation of the Ti–Al–Cr single crystal along the tensile loading direction (LD) in the inverse pole figure. **b**, Comparison between the Ti–Al–Cr alloy and other shape-memory alloys in terms of density, critical strength and specific strength at room temperature^3,15,23,24^. **c**, In situ strain fields obtained by means of the DIC method at different deformation levels during tensile loading–unloading testing, where P represents the untransformed parent phase region and M represents the martensite phase region. The white arrow indicates the direction of the moving transformation front. Scale bar, 2 mm.

**Extended Data Fig. 1:**
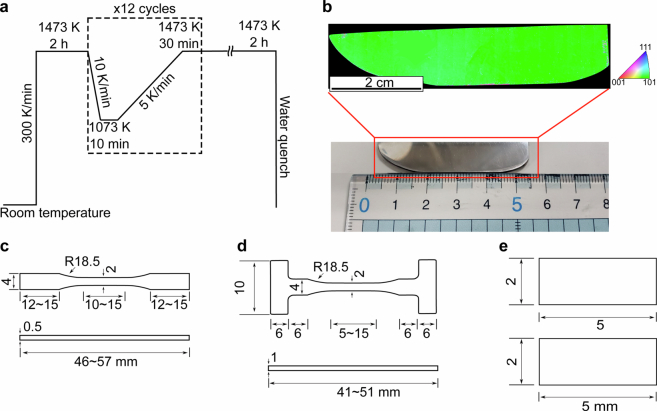
Cyclic heat treatments to produce large single-crystal Ti–Al–Cr specimens for mechanical tests. **a**, The cyclic heat treatment process. **b**, A large single crystal obtained by the cyclic heat treatment. The inverse pole figure map, illustrating the crystal orientation in the nominal direction, was shown. **c**, The specimen dimension for tensile testing at various temperatures. **d**, The specimen dimension for in situ neutron diffraction measurements. **e**, The specimen dimension for compression tests.

**Extended Data Fig. 2:**
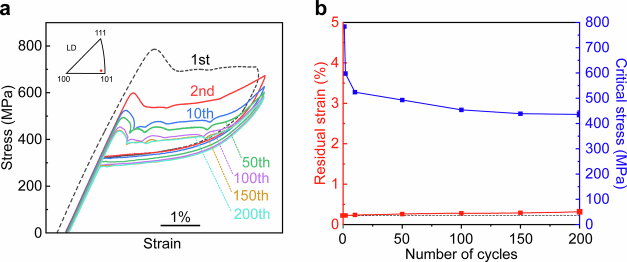
Cyclic stability of the superelasticity at room temperature. **a**, Stress–strain curves of 200-cycle loading–unloading tensile tests, the upper inset shows the orientation of the Ti–Al–Cr single crystal along tensile loading direction (LD) in the inverse pole figure. **b**, Residual strain and critical stress of the forward transformation as a function of number of cycles.

**Extended Data Fig. 3:**
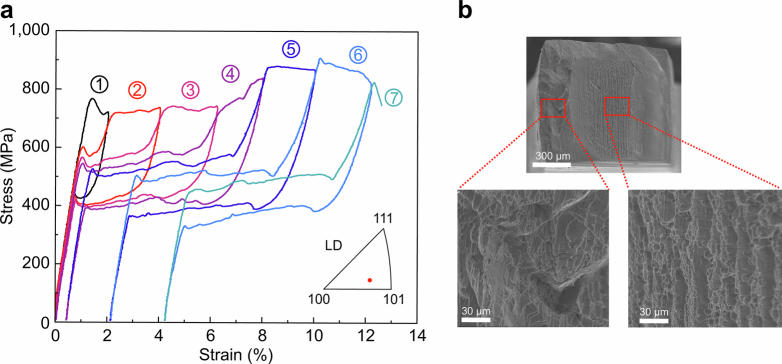
Mechanical response for the Ti–Al–Cr alloy to the tensile deformation until specimen fracture. **a**, The incremental stress–strain curves obtained at room temperature; the lower inset shows the orientation of the Ti–Al–Cr single crystal along the tensile loading direction (LD) in the inverse pole figure. **b**, Typical fracture surfaces of the Ti–Al–Cr alloy, where the dimple microstructure can be observed.

The chemical composition of the newly developed alloy is Ti–20Al–4.75Cr in atomic percent, and is hereafter referred to as Ti–Al–Cr. This alloy shows an excellent grain-coarsening ability through abnormal grain growth, enabling the preparation of large single crystals up to several centimetres for mechanical tests (Extended Data Fig. 1). Figure 1a shows the stress–strain curves obtained by means of uniaxial tensile loading–unloading testing at room temperature (297 K) for a single crystal. The flag shape of the complete curve loop indicates a superior superelasticity. This alloy achieves a recoverable strain of more than 7.3%, which is comparable to that of commercial Ni–Ti alloys. Notably, this recoverable strain is impressive for a Ti-based shape-memory alloy, being twice that of benchmark Ti–Nb-based alloys and 1.2 times larger than the previously recorded Ti–Zr–Nb–Sn alloys^15,16^. This superelasticity also showcases durable functionality, with a functional fatigue resistance exceeding several hundreds of the loading–unloading cycles (Extended Data Fig. 2). Figure 1a shows that the critical stress breaking out in the linear elastic region, which is analogous to the yield stress observed in steels, reaches roughly 800 MPa. This high strength combined with a low density of 4.36 × 10^3^ kg m^−^^3^ underscores the exceptional strength-to-mass characteristics inherent to Ti-based alloys. In Fig. 1b, we compare the critical strength and density of the Ti–Al–Cr alloy at room temperature with commercial Ni–Ti (ref. ^3^), conventional Ti-based^15,16,23^ and Mg-based shape-memory alloys^24^. The Ti–Al–Cr alloy achieves a remarkable specific strength of 185 × 10^3^ Pa m^3^ per kg: about twice that of previous lightweight shape-memory alloys. In addition to its remarkable superelasticity, the Ti–Al–Cr alloy also shows good ductility at room temperature, boasting an ultimate tensile strength of more than 900 MPa and a total elongation exceeding 12%, as revealed by the uniaxial tensile test conducted until specimen fracture (Extended Data Fig. 3).

We further used in situ surface observations and the digital-image-correlation (DIC) technique to characterize the meso-scale microstructure changes and the deformation coordination during the superelastic deformation. The in situ optical observation reveals the generation and subsequent propagation of a plate-like morphology during the loading process (Supplementary Video 1). Such surface relief is characteristic of stress-induced martensitic transformation, which is identified as the cause of superelasticity in the Ti–Al–Cr alloy. Figure 1c shows the DIC strain field images captured at different deformation states on tensile loading; the specimen initially undergoes minor homogeneous deformation, indicative of the elastic region, followed by the formation and steady propagation of a macroscopic band. This sequence corresponds to the nucleation and growth of single-variant martensite, culminating in a spontaneous strain field of 7.3%. This peak strain value aligns with the recoverable strain observed in Fig. 1a. The subsequent sequential DIC images from the unloading process show complete recovery in strain fields, suggesting excellent recoverability in superelasticity.

## Structural characterizations

**Fig. 2:**
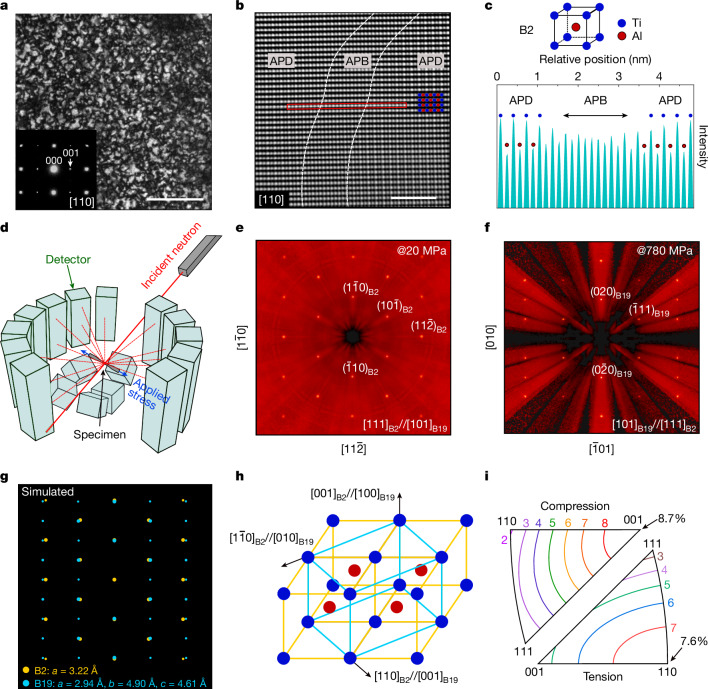
Microstructure observations and in situ neutron diffraction characterizations for a Ti–Al–Cr single crystal. **a**, TEM observation of the parent phase taken along the [110]_B2_ zone axis. **b**, STEM observation of the parent phase, viewed along the [110]_B2_ zone axis. **c**, Profile of the intensity along the red line as indicated in STEM imaging, where antiphase domain (APD) and APB can be distinguished. **d**, Schematic of the in situ neutron diffraction measurement setup. **e**, Two-dimensional neutron diffraction patterns collected before tensile loading, showing the B2 structure. **f**, Two-dimensional neutron diffraction patterns collected during tensile loading, showing the B19 structure. **g**, Reconstructed overlapped neutron diffraction patterns of both phases. **h**, Lattice correspondence between B2 parent phase and B19 martensite phases determined from the in situ neutron diffraction results. **i**, Calculated orientation dependence of transformation strain under tension and compression. Scale bars, 200 nm (**a**) and 2 nm (**b**).

To understand the atomic origin of the exceptional superelasticity of the Ti–Al–Cr alloy, we performed a structural analysis of the responsible phase transformation. Figure 2a shows the microstructural characterization results for the parent phase obtained using dark-field transmission electron microscopy (TEM) observations. Long-range ordering, specifically a B2-type ordered BCC structure in the form of nanodomains, is evident, as indicated by the (001) superlattice spot in the electron diffraction pattern. These B2 nanodomains have an average diameter of roughly 15 nm. This suggests that the order–disorder transformation from the A2 to B2 structure occurs during quenching^21^. The B2 nanodomains are separated by disordered antiphase boundaries (APBs), as revealed by the high-angle annular dark-field scanning transmission electron microscopy (STEM) images, shown in Fig. 2b. The APB is slender, with a width of roughly 2 nm. In addition, atomic imaging provides chemical information at atomic scale because the intensity of atomic column varies with atomic number. The intensity profile of a line crossing an APB is shown in Fig. 2c. The contrast in intensity between adjacent superlattice sites is distinctly observable in ordered nanodomains, whereas the periodicity of intensity becomes markedly less pronounced within the disordered APB region. Such a long-range-ordered structure in the parent phase, which differs from that of conventional disordered Ti-based shape-memory alloys, may render the Ti–Al–Cr alloy more resistant to dislocation-stimulated plastic deformations owing to the ordering strengthening effect^25^.

Determining the crystal structure of the stress-induced martensite phase is challenging because the responsible martensite phase is only stable under stress, which causes experimental difficulties. To address this, we used an in situ neutron diffraction method to characterize the crystal structure of the martensite phase under stresses. The experimental setup is schematically shown in Fig. 2d. Here, neutron diffraction patterns were collected simultaneously using time-of-flight Laue techniques as the single-crystal specimen was stretched at room temperature. The two-dimensional reciprocal lattices of the Ti–Al–Cr crystal under different load conditions were reconstructed by identifying the best-fitted unit cell based on the collected Laue patterns. The diffraction patterns captured and analysed for the specimen both in its untransformed parent phase and in the martensite phase under higher stresses are shown in Fig. 2e and f, respectively. The lattice parameter of the parent phase with a B2 structure was determined to be *a* = 3.22 Å. The stress-induced martensite phase was found to have an ordered orthorhombic (B19) crystal structure, with lattice parameters of *a* = 2.94 Å, *b* = 4.90 Å and *c* = 4.61 Å. Note that the lattice parameters of the martensite phase were determined under a stress of around 780 MPa. The simulated diffraction patterns of both the parent and the martensite phases, as shown in Fig. 2g, were reconstructed from the neutron diffraction patterns, and the lattice correspondence was determined, as shown in Fig. 2h. By applying a lattice deformation theory involving the lattice correspondence and lattice parameters^15^, the orientation-dependent transformation strain for the B2 → B19 transformation could be computed and is shown in Fig. 2i. The maximum recoverable transformation strain is calculated to be roughly 8.7% under <001> compression and 7.6% under <110> tension. These transformation strains occur between the parent phase, which is nearly stress free, and the martensite, which is under stress. The experimentally observed maximum recoverable tensile strain in Fig. 1a aligns with the calculated results. Hence the pronounced superelastic deformation in Ti–Al–Cr alloy can be confirmed to result from such an B2 → B19 martensitic transformation between ordered structures. Although this transformation is similar to that observed in Cu-added Ni–Ti-based alloys^26^, it is new for Ti-based alloys.

## Superelasticity at extensive temperatures

**Fig. 3:**
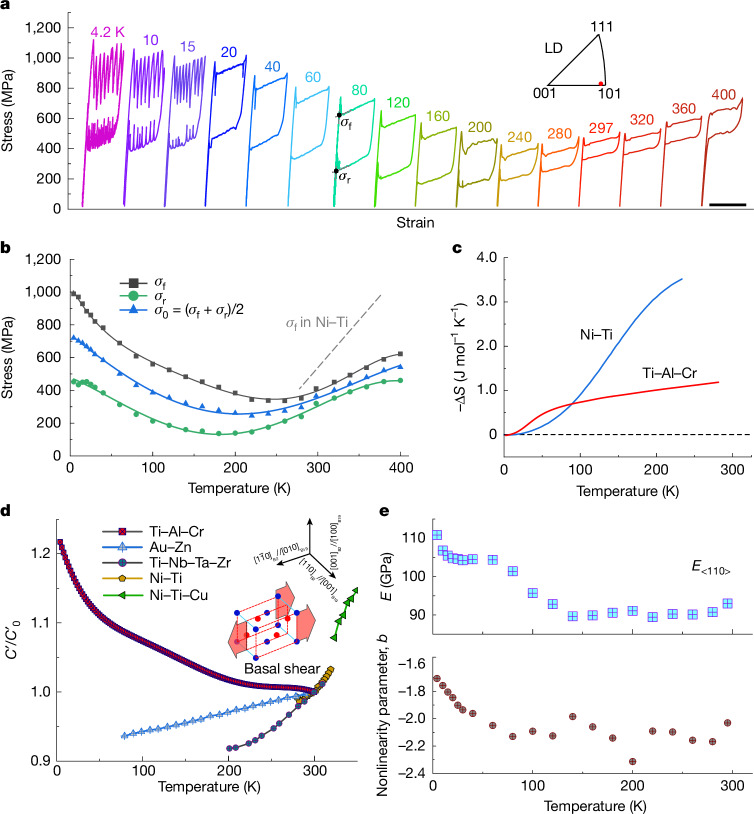
Ti–Al–Cr alloy showing superelastic behaviour across an extensive temperature range. **a**, A series of stress–strain curves obtained by means of tensile tests at various temperatures for a near-<110> single-crystal specimen. The upper inset shows the orientation of the Ti–Al–Cr single crystal along the tensile LD in the inverse pole figure. **b**, The corresponding temperature dependence of transformation stresses. **c**, Temperature dependence of the entropy change Δ*S* between the parent and martensite phases for the Ti–Al–Cr alloy, derived from the specific heat capacity of both phases, with that of Ni–Ti is shown for comparison^6^. **d**, Relative change in the shear modulus *C*′ compared to *C*′_0_ versus temperature for the Ti–Al–Cr alloy juxtaposed with the corresponding values of other typical shape-memory alloys^3,33–36^. Here, *C*′_0_ refers to the value of *C*′ at 300 K. **e**, Temperature dependence of Young’s modulus along the near-<110> orientation and the strain-expanded nonlinearity parameter *b*(=*D*/*E*) derived from the tensile responses at various temperatures. Scale bar, 5%.

**Extended Data Fig. 4:**
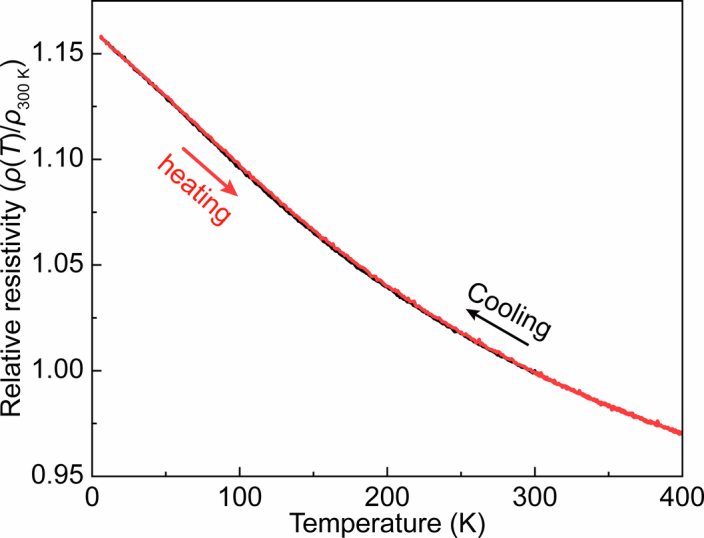
Relative change in electrical resistivity with respect to the value at room temperature as a function of temperature. There was no visible hysteresis loop or abrupt change during the cooling and heating processes, indicating the absence of a thermally induced martensitic transformation.

**Extended Data Fig. 5:**
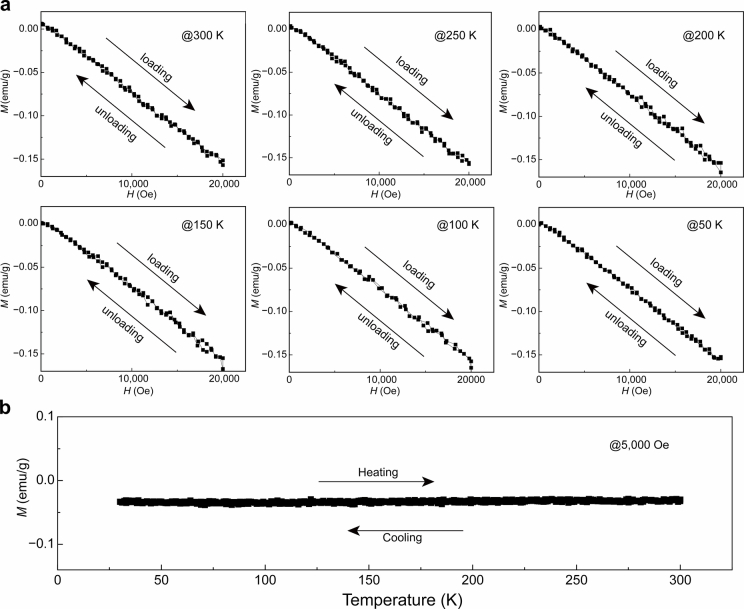
Magnetic properties of the Ti–Al–Cr alloy. **a**, The magnetization (*M*) versus the applied external magnetic field (*H*) at several selected temperatures, *T* = 300, 250, 200, 150, 100 and 50 K. The *M*–*H* curves show that the Ti–Al–Cr alloy is most likely a diamagnetic material, which means the material hardly repulses the magnetic field. **b**, The *M*–*T* curve, which indicates that there is no magnetic transition across the temperature range tested.

Unlike most shape-memory alloys that show superelasticity at around room temperature, the Ti–Al–Cr alloy does not undergo thermally induced martensitic transformation, even after significant cooling (Extended Data Fig. 4). Intrigued by this unusual behaviour, we explored its superelasticity at various temperatures. Figure 3a shows the stress–strain curves for the alloy obtained by means of loading–unloading uniaxial tensile tests targeting a strain of 5% across different temperatures. The Ti–Al–Cr alloy demonstrates complete superelastic recovery over a broad temperature range, that is, from 4.2 to 400 K. This suggests an operational temperature window for the alloy of at least 396 K, which is roughly five times that of commercial Ni–Ti alloys typically operating between 273 and 353 K (ref. ^3^). At temperatures below 20 K, pronounced serrations in the stress–strain curves can be observed, a phenomenon commonly seen in metals during cryogenic deformation^27^. This is probably caused by dynamic pinning–depinning interactions between martensite nucleation, variant growth and pre-existing defects, such as dislocations, in the present alloy. This serration effect may be mitigated by enhancing lattice compatibility of the martensitic transformation through compositional adjustment. It is evident that the anomalous temperature dependence of transformation stresses plays an important role for the ultra-broad operational temperature window. The extrapolated critical stresses of the forward (*σ*_f_) and the reverse (*σ*_r_) transformations, as well as the equilibrium stress (*σ*_0_) being the average of the previous two, are plotted against temperature in Fig. 3b, with the results for Ni–Ti for comparison^3,20^. Above roughly 200 K, the transformation stresses show a positive temperature dependence similar to most conventional shape-memory alloys. However, a negative temperature dependence is observed at lower temperatures. This phenomenon is the key to achieving a broader operational temperature window to prevent the loss of superelasticity when the transformation stresses drop below a certain level. The inverse temperature dependence of transformation stresses has been reported in a few ferromagnetic Co- and Fe-based shape-memory alloys and has been explained in terms of magnetism contribution to the relative phase stability between parent and martensite phases at low temperatures^5,28^. However, such a magnetism-related explanation seems inapplicable to our Ti–Al–Cr alloy given its non-magnetic nature (Extended Data Fig. 5).

## Analysis and discussion

**Extended Data Fig. 6:**
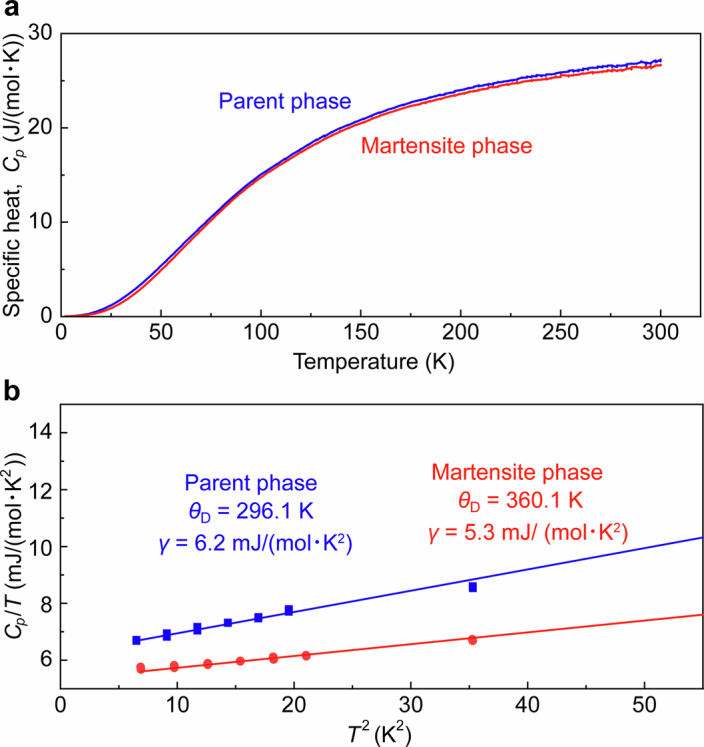
Thermodynamic analysis based on the specific heat data obtained by experiments. **a**, Specific heat of parent phase (Ti–20Cr–4.75Cr) and martensite phase (Ti–20Cr−4.25Cr) as a function of temperature. **b**, The comparison of the Debye temperature *θ*_D_ and the electronic specific heat coefficient *γ* between the two phases. The *C*_*p*_/*T* vs *T*^2^ was plotted and fitted for determining *θ*_D_ and *γ*.

The Clausius–Clapeyron relation is commonly used to assess the temperature (*T*) dependence of transformation stresses (*σ*_0_) in shape-memory alloys, focusing on the entropic aspect from a thermodynamic perspective^29^:

1 $$\frac{{\rm{d}}{{\sigma }}_{0}}{{\rm{d}}T}=-\frac{\Delta S}{{\varepsilon }{V}_{{\rm{m}}}},$$

where Δ*S* is the molar entropy difference between the parent and martensite phases, *ε* is the strain caused by the phase transformation and *V*_m_ is the molar volume. The Δ*S* of the Ti–Al–Cr alloy as a function of temperature, which is derived from the specific heat capacity of both phases at constant pressure (Supplementary Information and Extended Data Fig. 6), is shown in Fig. 3c. As the temperature approaches 0 K, the absolute value of Δ*S* reduces but does not change its sign. This was further confirmed by an elastocaloric measurement, which assessed the near-adiabatic temperature change of the specimen on reverse phase transformation at several representative temperatures (Supplementary Information). According to equation (1), the fact that Δ*S* maintained a consistent sign across temperature variations means that the transformation stress does not invert as temperature decreases because *ε* and *V*_m_ are always positive. However, this finding contrasts with the observed stress–strain behaviours shown in Fig. 3b.

**Extended Data Fig. 7:**
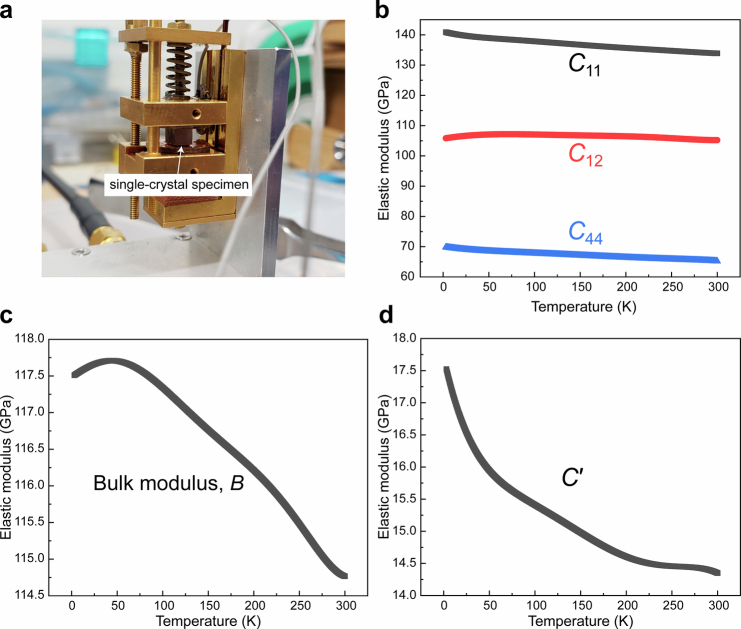
Temperature dependence of the elastic moduli of the parent phase. **a**, Experimental setup for determining elastic constants using the ultrasonic pulse-echo method. **b-d**, The elastic constants of *C*_11_, *C*_12_, *C*_44_, bulk modulus *B* and shear modulus *C*′ as a function of temperature, respectively.

**Extended Data Fig. 8:**
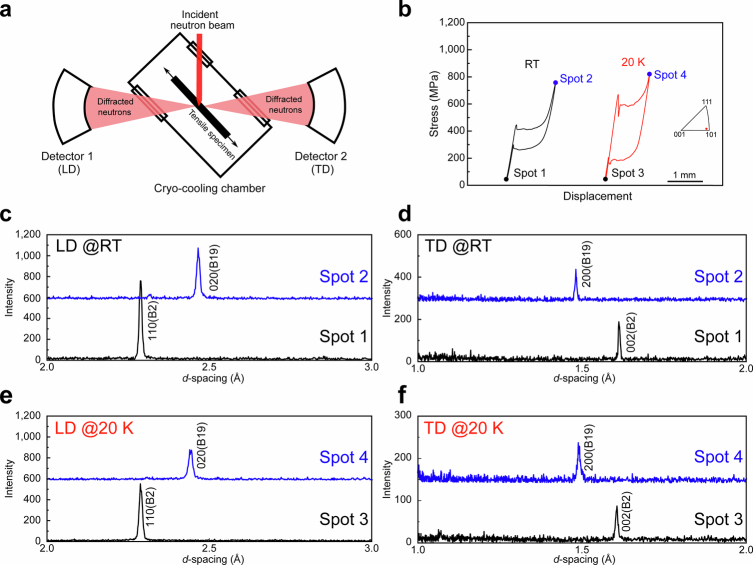
In situ neutron diffraction measurements at room temperature (RT) and 20 K conducted at TAKUMI. **a**, Schematic of the experimental setup. Neutron diffraction patterns along both the loading direction (LD) and the transverse direction (TD) can be obtained at the same time during uniaxial mechanical testing. **b**, Stress–displacement curves measured at RT and 20 K, respectively. **c-f**, In situ neutron diffraction patterns from LD and TD detectors obtained at both RT and 20 K, with the specimen held at positions indicated as spot 1, spot 2, spot 3, and spot 4 in **b**. The comparison of diffraction patterns at RT and 20 K suggests that the crystal structures of the parent phase and the martensite phase remain essentially unchanged at the tested temperatures.

The discrepancy between the practical stress–strain responses and the Clausius–Clapeyron-relation-based analysis underscores the importance of considering the lattice dynamics of the parent phase, especially the local mechanical instability of the crystal lattice^30,31^. Emphasis was placed on the lattice distortion of the parent phase, which is the state before the initiation of stress-induced martensitic transformation. This lattice distortion, which involves lattice dynamics, can be evaluated using the elastic modulus^32,33^. We measured the shear modulus *C*′ of the parent phase as a function of temperature, with the results shown in Fig. 3d and Extended Data Fig. 7. The shear modulus *C*′, which serves as an indicator of resistance to $\{110\}\langle 1\bar{1}0\rangle$ basal shear, plays a pivotal role in assessing lattice instability. This instability is instrumental in the formation of basal-plane-based martensite, as in the present case of the B2 → B19 transformation. Unlike conventional beta-phase shape-memory alloys^3,33–36^, the *C*′ of Ti–Al–Cr alloy shows a negative temperature dependence. On cooling, *C*′ initially shows a mild increase around 200 K, followed by a rapid ascent below roughly 70 K. Notably, the *C*′ value at 4.2 K is 22% higher than that at room temperature, indicating that the BCC lattice of the parent phase becomes increasingly resistant to shear deformation. Consequently, greater stress is required to achieve a certain degree of lattice distortion towards lattice disintegration. This negative temperature dependence of *C*′, which is anomalous for beta-phase shape-memory alloys, is typical for most normal BCC elements or alloys and probably results from internal electronic or ionic interactions in metallic bonding^37,38^. This is also considered to be the reason for the prevention of thermally induced martensitic transformation in the present alloy from a lattice dynamics perspective, which plays a role in martensite nucleation (Supplementary Information). Note that the negative temperature dependence of transformation stresses in a narrow temperature range in certain disordered Ti-based shape-memory alloys has been reported to be correlated with phase decomposition or separation in the parent phase^39,40^, but neither was observed in our alloy (Supplementary Information). The martensite phase also maintained its crystal structure across the studied temperature range (Extended Data Fig. 8), which excludes the possibility of different phase transformation. Furthermore, the influence of lattice anharmonicity, which also affects the mechanical stability of the crystal lattice in the parent phase, is considered^41–43^. We used the strain-expanded nonlinearity parameter *b*(=*D*/*E*) (ref. ^42^), which is the ratio of incipient Young’s modulus, *E*, to third-order modulus, *D*, determined by means of nonlinear least-squares fitting of the measured tensile elastic response to gauge lattice anharmonicity at different temperatures, as shown in Fig. 3e. The incipient Young’s modulus generally increases with decreasing temperature, in accordance with the negative temperature dependence of *C*′. The lattice anharmonicity characterized by *b*, remains relatively unchanged down to about 100 K during cooling and weakens with further cooling, signifying the enhanced mechanical stability of the crystal lattice at lower temperatures. Although the discrepancy between experimental observations in stress–strain responses and thermodynamic analysis cannot be entirely resolved, the temperature-dependent lattice instability suggested by the trends in temperature-dependent *C*′ and lattice anharmonicity is believed to be related to an increase in transformation stresses on cooling. This may contribute to the stabilization of crystal lattice of parent phase in low temperature region, that is, to the widening the operational temperature range for superelasticity. The classical Clausius–Clapeyron relation may not fully describe the negative temperature dependence of transformation stresses in a new class of shape-memory alloys that show a negative temperature dependence of the shear modulus *C*′, because of the temperature-dependent energy required to drive martensite nucleation (Supplementary Information). This could lead to the discovery of more shape-memory alloys with similar behaviour to the Ti–Al–Cr alloy.

## Summary and implications

**Fig. 4:**
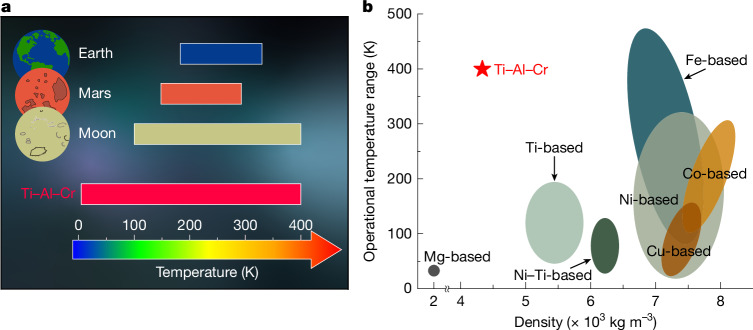
Comparison of superelastic temperature range and lightweight properties for various bulk shape-memory alloys. **a**, Superelastic temperature range of the Ti–Al–Cr shape-memory alloy versus temperature fluctuations of practical application environments including Earth, Mars and the Moon. **b**, Density versus operational temperature range of superelasticity for various shape-memory alloys^3–5,15,20,23,28,46–49^. The density of the alloys is measured value at room temperature.

**Extended Data Fig. 9:**
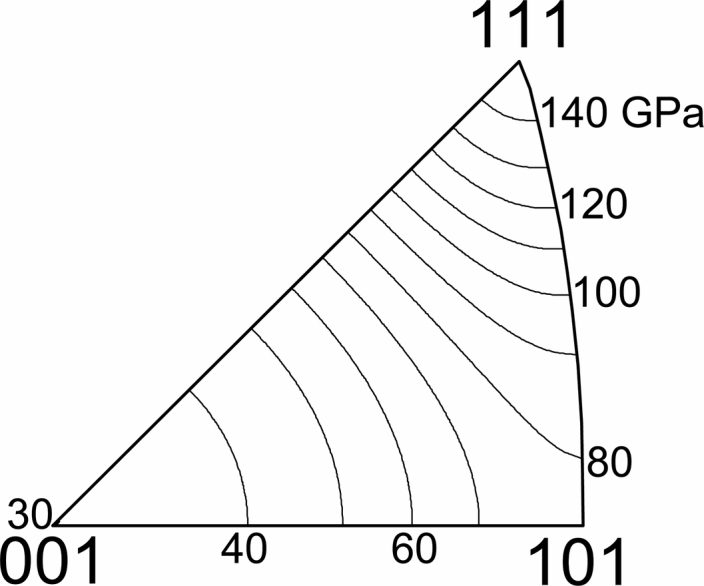
Orientation dependence of Young’s modulus for the Ti–Al–Cr alloy. The result is calculated by using experimentally determined elastic constants at room temperature.

This newly developed Ti–Al–Cr shape-memory alloy, a member of the lightweight yet strong, biocompatible and corrosion-resistant Ti alloy family, is promising for many applications because of its wide-temperature-range superelasticity. In interplanetary missions, such as Artemis I (ref. ^44^), creating metallic materials that are resilient to harsh conditions while retaining their functionality is a significant technological hurdle. For instance, the superelastic Ni–Ti tyres designed for the upcoming Moon and Mars missions have limitations in their operational temperature ranges^45^. As shown in Fig. 4a, our Ti–Al–Cr shape-memory alloy showing optimal superelastic behaviour with an operational window of about 400 K, combined with its high specific strength, is promising for applications for deep-space and deep-sea explorations. Compared with certain Fe-based shape-memory alloys that have a similarly broad operational temperature range or other shape-memory alloys^3–5,15,20,23,28,46–49^, the Ti–Al–Cr alloy excels in terms of lightweight property (Fig. 4b) and offers considerably higher resistance to functional fatigue. This enables safer and more energy-efficient applications. This Ti–Al–Cr alloy also has potential for medical applications owing to its low Young’s modulus of roughly 30 GPa along certain crystal orientation (Extended Data Fig. 9), which is lower than that of conventional Ti-based alloys and is close to that of human bones. Another important advantage of the proposed Ti–Al–Cr alloy system lies in its relatively low alloying element content, which offers potential cost reductions and sustainability benefits compared to conventional Ti-based shape-memory alloys, such as Ni–Ti or Ti–Nb. Both Al and Cr are more abundant and less costly than elements such as Ni and Nb (Supplementary Information), and the simpler composition of the Ti–Al–Cr system may lead to reduced environmental impact during extractive metallurgy and large-scale production^50^. With several potential applications, as well as the capability for mass production using existing metallurgical manufacturing processes developed for Ti-64, the lightweight yet strong Ti–Al–Cr shape-memory alloy opens up new possibilities for advancing the study and use of lightweight multifunctional materials.

## Methods

### Specimen preparation

Ingots of Ti–20Al–4.75Cr (at%) were prepared by arc-melting using high-purity elements of Ti, Al, Cr in an argon atmosphere, with a piece of Ti getter used to prevent potential oxygen contamination. The ingots were remelted at least five times to ensure composition homogenization. Ingots with less than 0.1% weight loss after arc-melting were subjected to hot rolling at 1,473 K with a 60% thickness reduction. The specimens were cut out and ground to remove surface contamination layer, then wrapped in Mo foils and sealed in argon-backfilled quartz tubes before heat treatments. After cyclic heat treatment as described in Extended Data Fig. 1, the specimens were quenched in water and aged at 473 K for 1 h. The oxygen content of the specimens before mechanical testing were precisely determined by the inert gas fusion-infrared absorption method, yielding 0.095% in weight, which is low, placing it within the range of commercial Grade 1 titanium alloys. Large single-crystal specimens with desired orientation along length direction were prepared by electrical discharge machining. Specimen surfaces were mechanically ground and polished first, and then chemo-mechanically polished with a colloidal silica suspension.

### Mechanical tests

To determine the strength, ductility and maximum recoverable strain, dog-bone-shaped single-crystal specimens with a gauge dimension of 10 × 2 × 0.5 mm was stretched along near <110> orientation by incremental cyclic loading–unloading tensile tests using a universal tensile tester (Shimadzu AG-X 10 kN) at room temperature. A contactless video extensometer was used to accurately measure the strain. The strain rate was fixed at 5 × 10^−4^ s^−1^.

To characterize the superelastic behaviour at various temperatures, tensile tests were performed at temperatures ranging from 4.2 to 300 K using a customized tester (Instron 5982) combined with a cryogenic system and from 300 to 400 K using a universal testing machine (Shimadzu AG-X 10 kN) equipped with a thermostatic chamber. Another dog-bone-shaped single-crystal specimen oriented along near <110> with a gauge dimension of 13 × 2 × 0.5 mm was used. The specimen was first mechanically trained at 300 K for ten cycles to achieve a stable superelasticity before being cooled down to 4.2 K. Then it was mechanically tested during the heating process. Mechanical tests were conducted three times to confirm the repeatability of superelastic behaviour at each temperature point. The strain was measured using a clip-on extensometer. The total tensile strain was fixed at 5% throughout the tests.

### Microstructure observations and crystal structure characterizations

The determination of crystal orientation and observation of fracture surface were performed in a field-emission scanning electron microscope (JEOL JSM-7800F) equipped with an electron backscattered diffraction detector. TEM (JEOL JEM-2100HC) and STEM (JEOL JEM-ARM200F) were also used to observe the microstructure. The TEM and STEM specimens were mechanically polished to a thickness of 80 μm and electropolished in a twin-jet polishing machine (Struers TenuPol-5) using a solution with 6% perchloric acid, 34% ethylene glycol and 60% ethanol (vol.%) at 253 K. X-ray diffraction measurements for evaluation of crystal structure were conducted on an X-ray diffractometer (Rigaku SmartLab 3 kW) with Cu Kα radiation.

### In situ DIC measurements

The in situ DIC technique was used to study the strain field evolution during the superelastic deformation at room temperature. The optical imaging was captured using a high accuracy microscope (Keyence VHX-8000). The uniaxial tensile test on a bulk <110> single-crystal specimen, with a gauge dimension of 10 × 2 × 0.5 mm, was conducted on a mini tensile stage (Deben 2kN), during which optical videos were recorded for strain profile assessment in an open-access GOM Correlate software. The strain component along the tensile direction was used to calculate the local strain distribution through the gauge section of interest. The in situ tensile test was performed at a crosshead speed of 0.3 mm min^−1^ and the maximum tensile strain was stopped at the point at which the gauged part fully transformed into martensite phase.

### In situ neutron diffraction measurements

In situ neutron diffraction experiments to determine the crystal structures of the martensite phase were carried out using the Extreme Environment Single Crystal Neutron Diffractometer SENJU located at Materials and Life Science Facility (MLF) of Japan Proton Accelerator Research Complex (J-PARC)^51^. The single-crystal specimen is near-[110] oriented along the tensile loading direction. The high-flux neutron source, with a beam power of 800 kW, and large-area time-resolved neutron detectors have made it possible to achieve highly accurate single-crystal neutron diffraction measurements using time-of-flight Laue technique. In situ tensile loading and unloading tests at room temperature were performed on a bulk dog-bone shaped single-crystal specimen with a gauge dimension of 6 × 1.7 × 1.8 mm as shown in Extended Data Fig. 1. The specimen was aligned with the loading direction horizontal at angles of 45° and 90° (the positive rotating direction is counterclockwise from the top view) to the incident neutron beam at each static measurement point to collect enough data. The neutron diffraction data were collected for a duration of 3,600 s for two angles separately, at which the specimen was statically held at various strain levels during loading and unloading procedures. An incident neutron beam slit of *Φ*3 mm was used. The neutron diffraction data were analysed using STARGazer software^52^. The lattice parameters were determined by finding the *UB* matrices that indexed the maximum number of peaks with the minimum mismatch. Here, the *UB* matrix is a mathematical matrix relating the reciprocal lattice space, which is related to the inherent structure of the crystal and the reciprocal space defined on the instrument.

In situ neutron diffraction experiments aimed at elucidating the evolution of crystal structures during martensitic transformation at cryogenic temperatures were conducted at the Engineering Materials Diffractometer TAKUMI within the MLF at J-PARC. These experiments involved in situ tensile loading and unloading tests on a bulk, dog-bone shaped single-crystal specimen with a gauge dimension of 6 × 1.7 × 1.8 mm. The loading apparatus was positioned such that the loading axis was horizontal and oriented at a 45° angle to the incident neutron beam, as depicted in Extended Data Fig. 8a. An incident neutron beam slit of 5 × 5 mm and a pair of *Φ*3-mm radial collimators, were used. Neutron diffraction measurements were performed under tension, maintaining a constant stress during each measurement within the elastic region and a constant strain (crosshead displacement) in the superelastic region.

### Electric resistivity, magnetization and specific heat measurements

Electric resistivity was measured by using a four-probe method from 6 to 400 K in a physical properties measurement system (PPMS) (Quantum Design). Magnetization measurements up to 20 kOe were conducted from 50 to 300 K using the PPMS equipped with alternating current measurement system option. The specific heat of the parent phase and the martensite phase from 2 to 300 K was measured using heat capacity option of PPMS system, using the relaxation method. The martensite phase specimen was measured using a compressed Ti–20Al–4.25Cr single crystal in which a slightly lower Cr concentration makes residual martensite on unloading. The compressed specimen section, fully transformed into the martensite phase as confirmed by electron backscattered diffraction results, was cut out for specific heat measurement.

### Experimental determination of elastic constants at various temperatures

The elastic constants from 2 to 300 K were measured by the ultrasonic pulse-echo method using an ultrasonic option of the PPMS. The elastic constant *C* (GPa) can be obtained by the following equations:

2 $$C=\rho {v}^{2},$$

where $\rho$ is the density of material and *v* is the sound velocity transporting across a certain crystal orientation.

The temperature dependence of the sound velocity in Ti–20Al–4.75Cr single crystal was measured by the phase comparison method. The longitudinal velocities were measured for [001] orientation, corresponding to *C*_11_, and [110] orientation, corresponding to $\frac{1}{2}$ (*C*_11_ + *C*_12_ + 2*C*_44_), using two longitudinal wave transducers. The transverse velocity vertical to [001], corresponding to *C*_44_, was measured using a pair of transverse wave transducers. The elastic constants determined at room temperature are listed in Supplementary Table 1. The correctness of the PPMS ultrasonic measurement results for 85 to 300 K have been independently verified using transient grating spectroscopy^53^.

## Online content

Any methods, additional references, Nature Portfolio reporting summaries, source data, extended data, supplementary information, acknowledgements, peer review information; details of author contributions and competing interests; and statements of data and code availability are available at 10.1038/s41586-024-08583-7.

## Supplementary information

Supplementary Information This file contains Supplementary Discussion, Figs. 1–17, Table 1 and Refs.

Supplementary Video 1 In situ optical surface observation during the tensile testing of a Ti–Al–Cr dog-bone-shaped specimen at room temperature.

## Data Availability

The data that support the findings of this study are available from the corresponding authors upon reasonable request.
