# Supplementary material for: A lightweight shape-memory alloy with superior temperature-fluctuation resistance
Source: Nature. 2025 Feb 26;638(8052):965–71. doi: 10.1038/s41586-024-08583-7 (PMC11864968; doi:10.1038/s41586-024-08583-7)
Supplement: Supplementary file 1 — This file contains Supplementary Discussion, Figs. 1–17, Table 1 and Refs. [file 41586_2024_8583_MOESM1_ESM.pdf]

nature portfolio

https://doi.org/10.1038/s41586-024-08583-7

Supplementary information

A lightweight shape-memory alloy with superior temperature-fluctuation resistance

In the format provided by the authors and unedited

Supplementary Information for

A lightweight shape-memory alloy with superior temperature fluctuation resistance

Authors: Yuxin Song[1]†, Sheng Xu[1,2]†*, Shunsuke Sato[1], Inho Lee[1], Xiao Xu[1], Toshihiro Omori[1]*, Makoto Nagasako[3], Takuro Kawasaki[4], Ryoji Kiyanagi[4], Stefanus Harjo[4], Wu Gong[4], Tomáš Grabec[5], Pavla Stoklasová[5], Ryosuke Kainuma[1]*

Affiliations:

[1]Department of Materials Science, Graduate School of Engineering, Tohoku University, Aoba-yama 6-6-02, Sendai 980-8579, Japan.

[2]Frontier Research Institute for Interdisciplinary Sciences, Tohoku University, Aramaki aza Aoba 6-3, Sendai 980-8578, Japan.

[3]Institute for Materials Research, Tohoku University, Katahira 2-1-1, Sendai 980-8577, Japan.

[4]J-PARC Center, Japan Atomic Energy Agency, Tokai, Ibaraki 319-1195, Japan.

[5]Institute of Thermomechanics, Czech Academy of Sciences, Dolejškova 5, 182 00 Prague, Czech Republic.

† These authors contributed equally to this work

*Corresponding authors. Email: xu.sheng.a8@tohoku.ac.jp (S. X.); omori@material.tohoku.ac.jp (T. O.); kainuma@material.tohoku.ac.jp (R. Ka.)

This PDF file includes:

Supplementary Discussion
Supplementary Figures 1 to 17
Supplementary Table 1
Supplementary Video 1
Supplementary References

Supplementary Discussion

1. Evolution of entropy change during martensitic transformation at various temperatures

To evaluate the difference in the total entropy change ΔS between the parent and the martensite phases, we experimentally measured the specific heat for both phases.

At temperatures below 10 K, a linear relationship holds well for the relationship between C_p/T and T^2, and this can be written as [1]:

$$C_p \approx C_v = \gamma T + \beta T^3 \quad \text{(S1)}$$

where C_v is the specific heat at constant volume. Conventionally, the first and second terms on the right-hand side of Eq. (S1) are the electronic and lattice vibration contributions, respectively, where γ is the electronic heat capacity coefficient and β is related to the Debye temperature, θ_D. The apparent Debye temperature is estimated by:

$$\theta_D = \sqrt[3]{\frac{12\pi^4 R}{5\beta}} \quad \text{(S2)}$$

where R is the gas constant. From the measurement results and the use of Eq. (S1) and (S2), γ and Debye temperature of both phases were estimated. From the intercept on the vertical axis, the value of γ, is evaluated to be 6.2 mJ/(mol·K^2) for the parent phase and 5.3 mJ/(mol·K^2) for the martensite phase. The value of θ_D is determined to be 296.1 K for the parent phase and 360.1 K for the martensite phase. The higher value of γ and lower value of θ_D in the parent phase compared with those of the martensite phase were also reported for Ti–Ni and Ni–Mn–Ga alloys [2, 3].

The results of specific heat measurements are used to evaluate the entropy change using the following relationship:

$$\Delta S = S^{\mathrm{M}} - S^{\mathrm{P}} = \int_0^T \frac{C_p^{\mathrm{M}} - C_p^{\mathrm{P}}}{T} \mathrm{d}T \quad \text{(S3)}$$

where the letters P and M on the shoulder indicate the phase to which the entropy contribution belongs. The change in the sign of ΔS against temperature was not observed for the present Ti-Al-Cr alloy, as shown in **Fig. 3c**.

2. Experimental measurements of elastocaloric effect at various temperatures

To characterize the elastocaloric effect at various temperatures, compression tests were performed at temperatures ranging from 48 K to 296 K using a customized loading frame equipped with a cryo-cooling chamber. A single-crystal specimen oriented along <001> with a dimension of 5 mm × 2 mm × 2 mm was used. To experimentally measure the adiabatic temperature change, ΔT_{exp}, during unloading, the stress was slowly increased to induce martensitic transformation and then rapidly removed at a strain rate of 0.24 s^{-1}. The specimen temperature was measured with thermocouple wires spot-welded to the surface of the specimen. The ideal attainable adiabatic temperature change, $\Delta T_{\mathrm{ideal}}$, is given by:

$$\Delta T_{\mathrm{ideal}} = \frac{T \cdot \Delta S}{C_p} \quad \text{(S4)}$$

where C_p is the heat capacity at the testing temperature T, ΔS is the entropy difference between parent phase and martensite phase. The dissipation energy, ΔW, was evaluated from the hysteresis loop area of the stress-strain curve, thus, the irreversible temperature change, ΔT_{dis}, can be calculated as:

$$\Delta T_{\mathrm{dis}} = \frac{\Delta W}{2C_p} \tag{S5}$$

The $\Delta T_{\mathrm{ideal}}$ was calculated by subtracting the ΔT_{dis} from the ΔT_{exp} and the value of ΔS was derived from the Eq. (S4). The evaluated ΔS data are plotted in **Supplementary Fig. 9**.

3. Chemical Gibbs free energy difference versus Driving force required for martensite nucleation

Typically, in shape-memory alloys, the chemical Gibbs free energy difference between the parent and martensitic phases increases as the temperature is lowered below the chemically equilibrium temperature T_0. Once this difference reaches a critical value, it drives martensite nucleation, and the corresponding temperature is referred to as M_s point [4]. This process is schematically illustrated in **Supplementary Fig. 15a**.

The Gibbs free energy difference between the parent and martensite phases ($\Delta G_{\mathrm{chemical}} = G_{\mathrm{M}} - G_{\mathrm{P}}$) can be calculated by using the equation:

$$\Delta G_{\mathrm{chemical}} = \Delta H - T\Delta S \tag{S6}$$

where ΔH and ΔS are the enthalpy and entropy differences, respectively. The values of ΔH and ΔS can be derived from the specific heat difference, ΔC_p ($= C_p^{\mathrm{M}} - C_p^{\mathrm{P}}$), as:

$$\Delta H = \Delta H_0 - \int_0^T \Delta C_p \mathrm{d}T \tag{S7}$$

$$\Delta S = \Delta S_0 - \int_0^T \frac{\Delta C_p}{T} \mathrm{d}T \tag{S8}$$

where ΔH_0 and ΔS_0 are the enthalpy and entropy changes at 0 K, respectively. The ΔS_0 is assumed to be zero from the third law of thermodynamics. The value of ΔH_0 is typically derived from Eq. (S6) at the equilibrium temperature T_0, where $\Delta G_{\mathrm{chemical}} = 0$, or from first-principles calculations.

The temperature dependence of $\Delta G_{\mathrm{chemical}}$ for typical shape-memory alloys exhibiting thermally induced martensitic transformation is illustrated in **Supplementary Fig. 15b** as a black solid line. As the temperature approaches 0 K, the slope of the $\Delta G_{\mathrm{chemical}}$ versus T curve approaches zero, resulting in nearly constant $\Delta G_{\mathrm{chemical}}$ at cryogenic temperatures. Meanwhile, the driving force required for martensite nucleation is shown as a red dashed line, set to a constant value for simplification. Once black solid line and red dashed line intersect, martensite nucleation is triggered, and the corresponding temperature is referred to as the M_s point.

From a kinetic perspective, martensite nucleation is driven by a faulting process, and the energy required for this nucleation can be correlated to the shear elastic modulus, as proposed by G.B. Olson [5, 6]. The higher the shear elastic modulus, the more energy required for martensite nucleation. This can also be rationalized from Clapp's localized soft-mode theory of martensite nucleation [7, 8]. For most shape-memory alloys, the energy required for martensite nucleation

decreases with cooling, as schematically shown in **Supplementary Fig. 15c**, often accompanied by elastic modulus softening or phonon softening [9]. However, in the Ti-Al-Cr alloy, the elastic modulus (such as *C*') increases as temperature decreases, indicating enhanced mechanical stability of the parent phase's crystal lattice. This results in an increased energy for martensite nucleation (**Supplementary Fig. 15d**). The combination of these factors—enhanced lattice stability and a relatively small Gibbs free energy difference at lower temperatures—prevents the nucleation of martensite, leading to the absence of cooling-induced martensitic transformation and contributing to the increased transformation stress required for stress-induced martensitic transformation at lower temperatures.

4. Sustainability and cost of the proposed Ti-Al-Cr alloy

The urgent need to build a green society calls for sustainable and low-cost metallurgy, which suggests using earth-abundant and recyclable elements. Conventional shape-memory alloys such as Ni-Ti and Ti-Nb-Ta-Zr (TNTZ) often rely on costly alloying elements and involve environmentally challenging extractive metallurgy processes. In contrast, the Ti-Al-Cr alloy system we have developed is intentionally designed to minimize the alloying element content, relying on more abundant and lower-cost elements such as aluminum and chromium, which are more sustainably sourced, as shown in **Supplementary Fig. 16**. The simpler composition not only reduces material costs but also minimizes the environmental footprint associated with alloy production and processing. In addition, the dilute nature of our alloy system is expected to simplify large-scale production and processing, as fewer complex steps (e.g., precise control of expensive alloying elements) are required. Furthermore, we summarized the global warming potential for the production of selected lightweight shape-memory alloys and titanium alloys relative to their raw material costs in **Supplementary Fig. 17**, which further demonstrates that the Ti-Al-Cr alloy offers superior environmental sustainability and economic viability. The reduced reliance on critical raw materials, combined with the alloy's favorable mechanical properties, suggests that the Ti-Al-Cr system has the potential to offer both economic and environmental advantages when scaled up for practical applications.

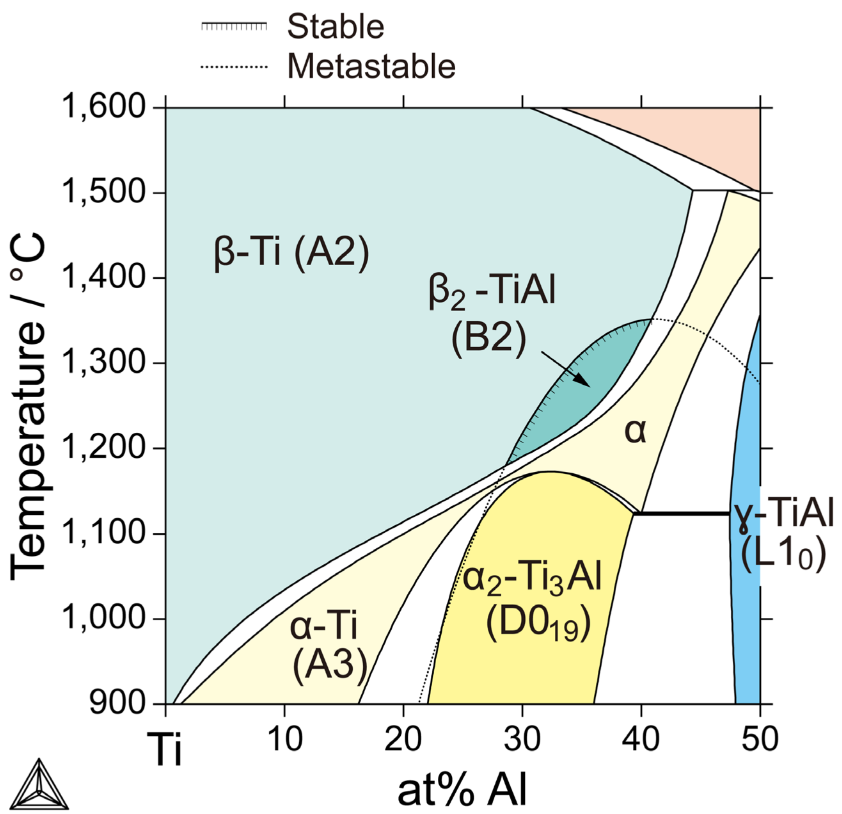

Supplementary Fig. 1 Binary phase diagram of Ti-Al system in the Ti-rich portion [10]. The A2/B2 order-disorder transition boundary is delineated by a broken line extending to the region near 20% Al.

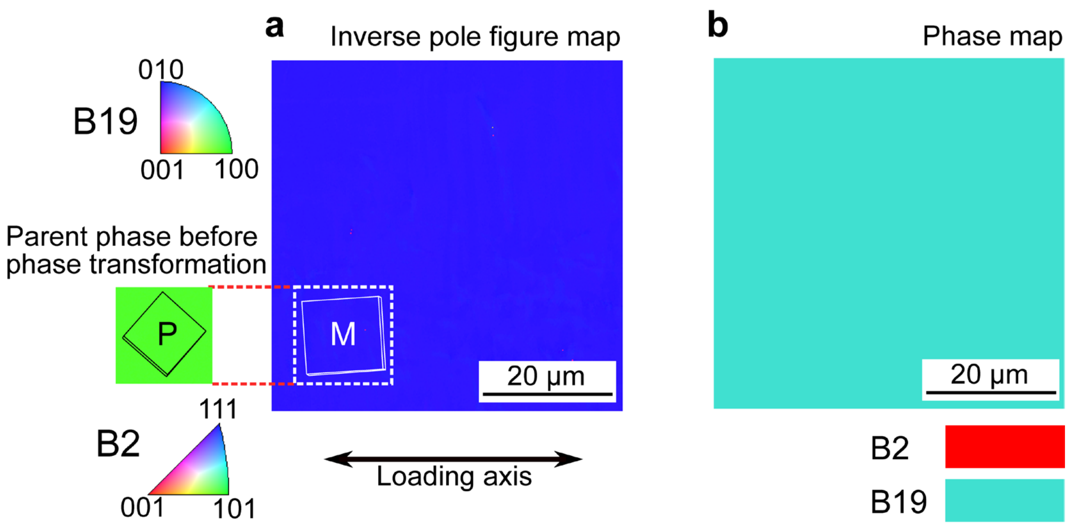

Supplementary Fig. 2 EBSD observation of a fully transformed martensite in a near-<110> single-crystal Ti-Al-Cr alloy under tensile loading. a, Inverse pole figure map in the loading direction. **b,** Phase map.

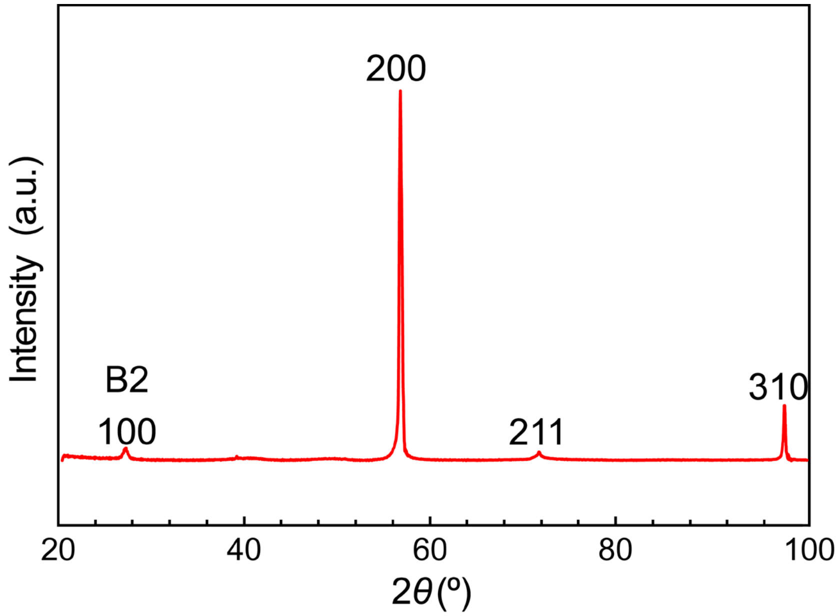

Supplementary Fig. 3 Structure characterization of a Ti-Al-Cr alloy by X-ray diffraction. The B2 single phase could be confirmed.

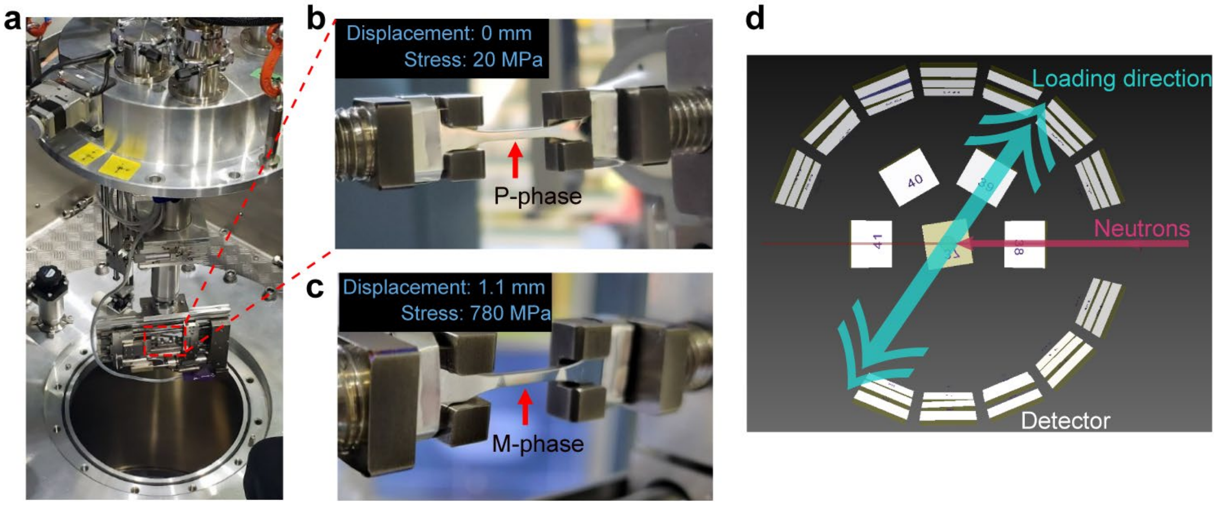

Supplementary Fig. 4 Experimental setup of SENJU for in situ neutron diffraction measurements. a, The tensile stage installed on a goniometer. **b-c**, Photos of the specimen before and after stress-induced martensitic transformation, respectively. **d,** Schematic diagram showing the uniaxial tensile loading direction with respect to the incident pulsed neutron beam.

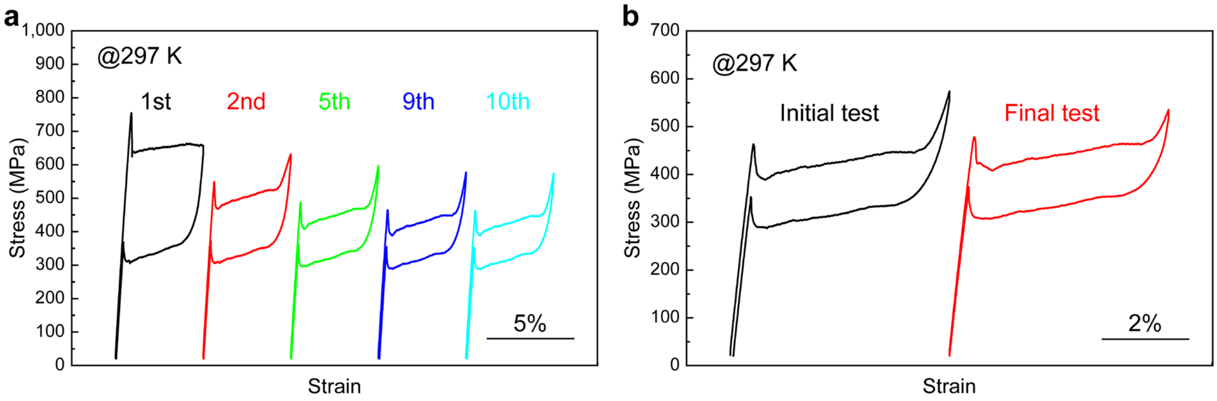

Supplementary Fig. 5 Stability of room-temperature superelasticity before and after the temperature-variation mechanical tests. a, Stress–strain curves in the training cycles, the transformation stresses become stable after 10-cycle training. **b,** Room-temperature stress–strain curves obtained for the trained specimen before and after mechanical testing at various temperatures, respectively. There is no noticeable difference between these two states.

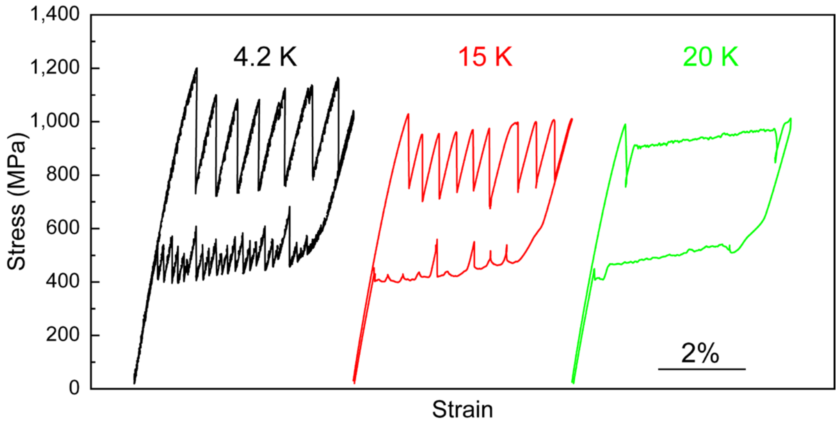

Supplementary Fig. 6 Serration phenomena during superelastic deformation at very low temperatures. Serration phenomena were observed in stress–strain curves obtained at temperatures lower than 20 K.

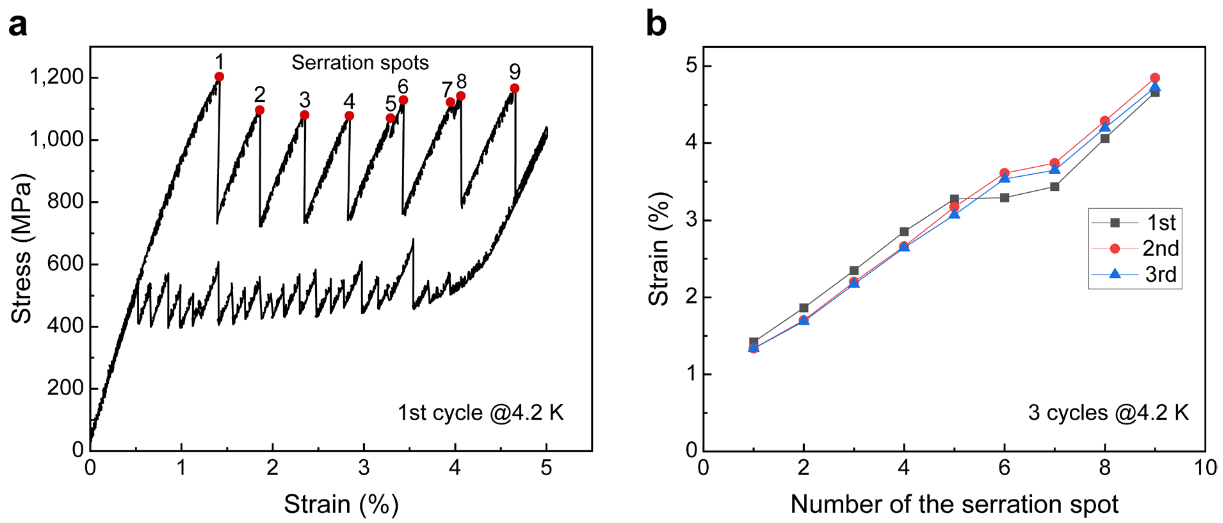

Supplementary Fig. 7 A brief quantitative analysis on the serration behaviors. a, A stress–strain curve by mechanical loading and unloading tests at 4.2 K, where the serration events during forward martensitic transformation are indicated and numbered. **b,** Comparison on the occurrence strain of each serration event across three consecutive loading–unloading cycles at 4.2 K. The serrated phenomenon is not random; serration events occur at nearly identical strains in the repeated stress–strain curves.

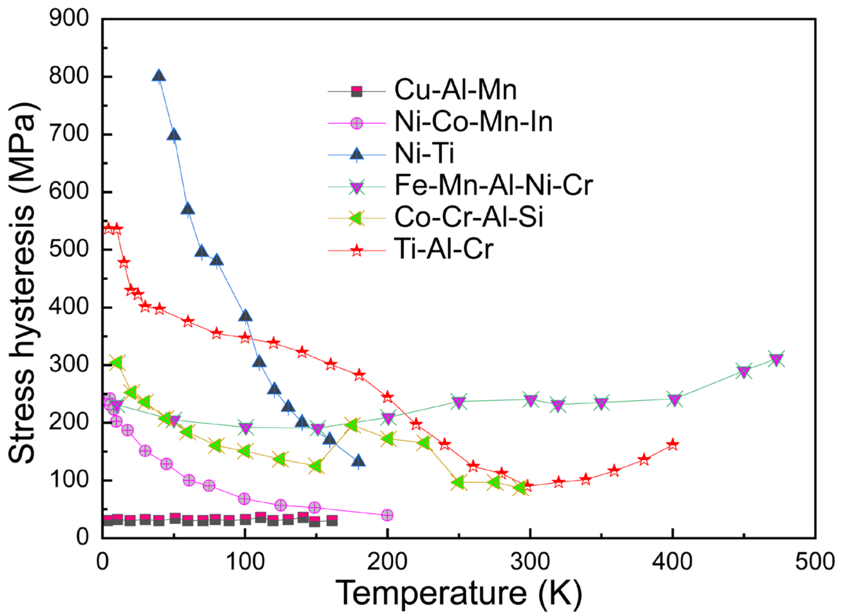

Supplementary Fig. 8 Stress hysteresis as a function of temperature. Though an increased stress hysteresis was identified at lower temperatures for the Ti-Al-Cr alloy, perfect superelastic recovery can still be achieved.

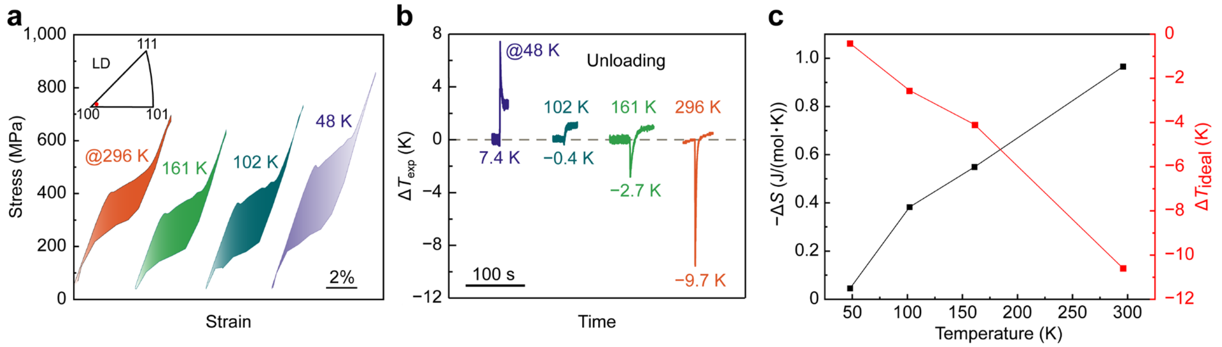

Supplementary Fig. 9 Temperature variation of elastocaloric properties. a, Compressive strain–stress curves are used to evaluate the elastocaloric effect at various temperatures, with dissipation energies derived from the areas of the hysteresis loops. The upper inset shows the orientation of the Ti-Al-Cr single crystal along the compression loading direction (LD) in the inverse pole figure. **b,** The experimental adiabatic temperature changes (ΔT_{exp}) during fast unloading are plotted as a function of temperature. **c,** The ideal adiabatic temperature change (ΔT_{ideal}), calculated by excluding the influence of dissipation energies from the ΔT_{exp}, is used to determine the entropy difference (ΔS) at various temperatures. No sign reversal is observed in ΔS.

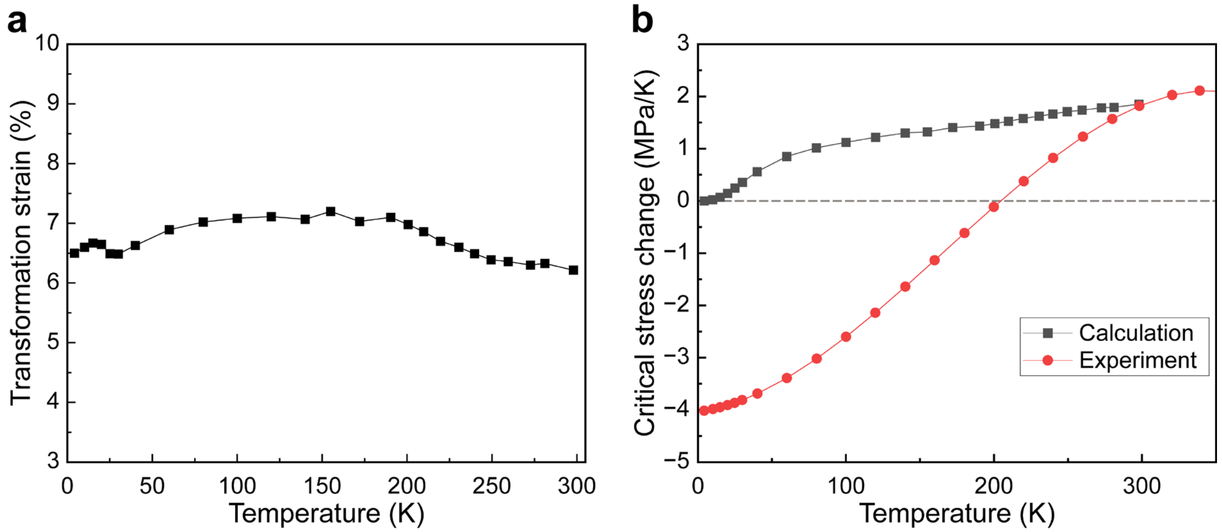

Supplementary Fig. 10 Temperature dependence of superelastic characteristics. a, Temperature dependence of maximum transformation strain observed during tensile test at various temperatures, the value of transformation strain is non-sensitive to temperature change. **b,** Temperature dependence of critical stress change ($d\sigma_0/dT$) obtained from tensile experiments (red dots), was compared with thermodynamic calculations derived from Clausius-Clapeyron equation using entropy difference obtained from the specific heat capacity (gray dots). The experimental temperature dependence of transformation stresses cannot be explained by solely considering thermodynamics.

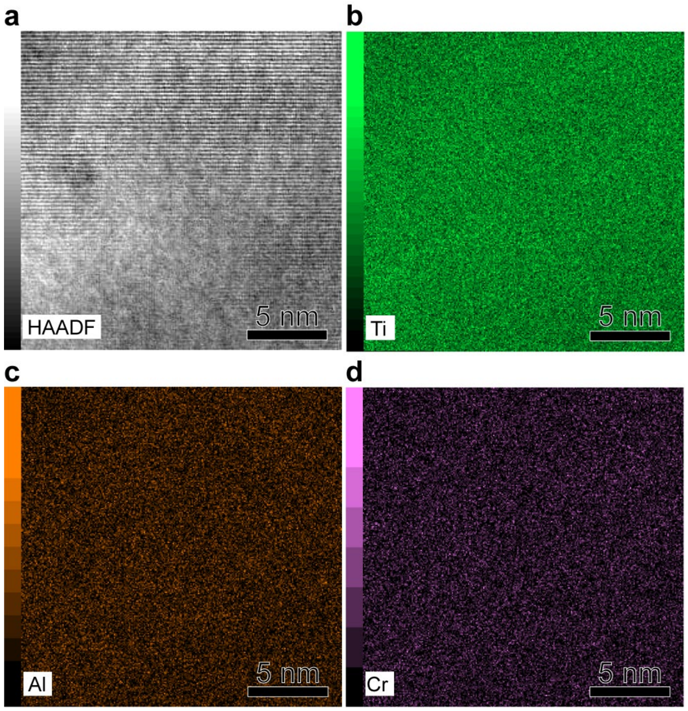

Supplementary Fig. 11 Distribution of the constituent elements at the nanoscale. a, The STEM-HAADF image from $[110]_{B2}$ zone axis. **b-d,** Elemental mapping obtained by energy-dispersive X-ray spectroscopy (EDS). There is no evidence of phase separation since the chemical compositions are evenly distributed at the atomic level.

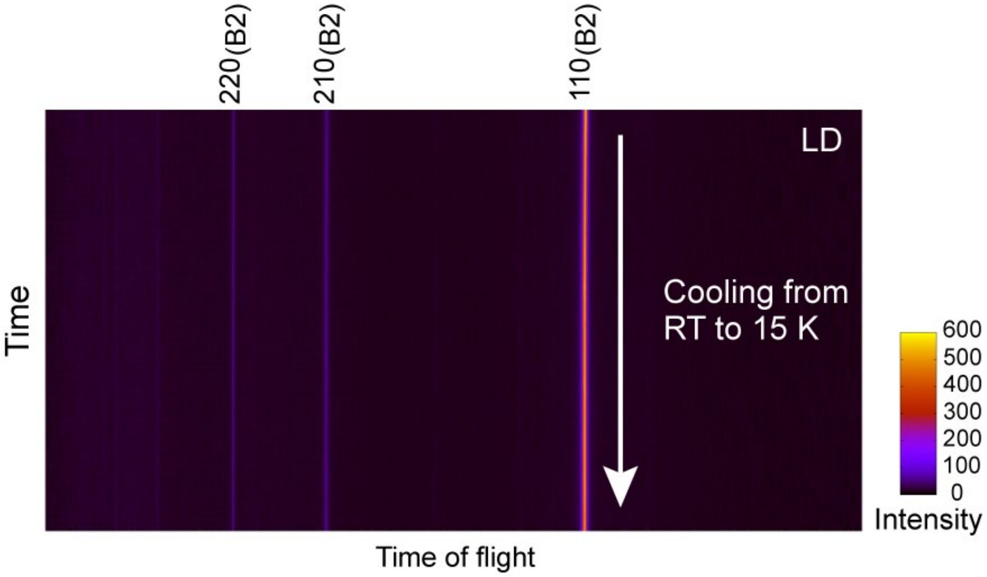

Supplementary Fig. 12 Evolution of the neutron diffraction pattern obtained by the loading direction (LD) detector during cooling from room temperature (RT). There is no applied stress, and the crystal structure of parent phase remains unchanged upon temperature change.

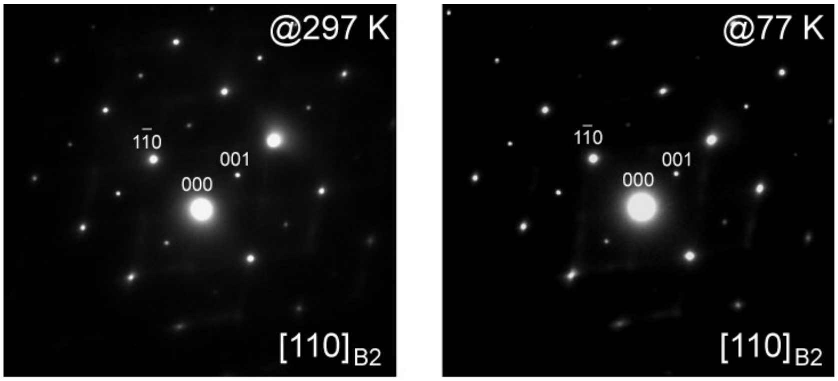

Supplementary Fig. 13 Electron diffraction patterns obtained by TEM. The temperature is 297 K and 77 K, respectively.

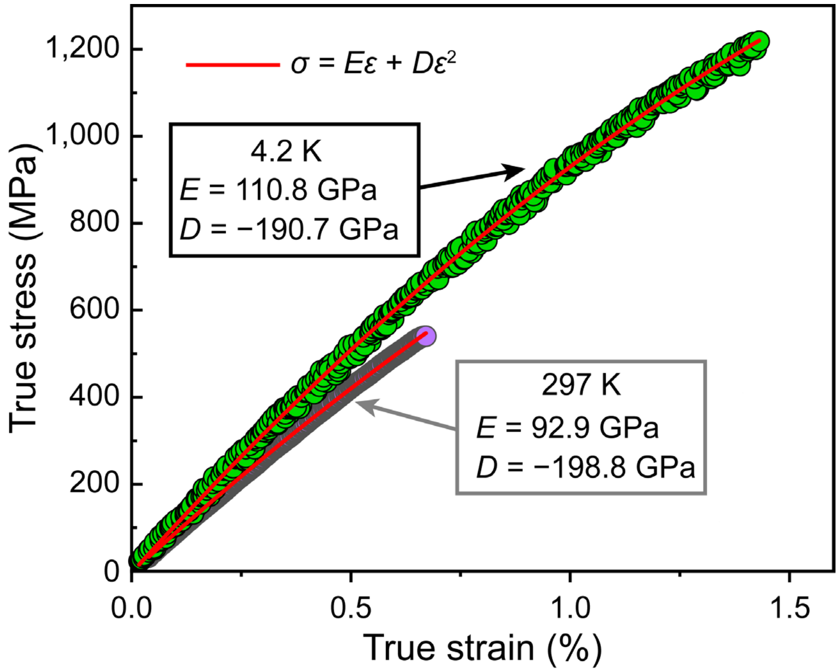

Supplementary Fig. 14 Typical true stress–strain curves in the elastic region. The loading stress–strain curves for the near-<110> tensile testing and the fitted curves at 4.2 K and 297 K, respectively.

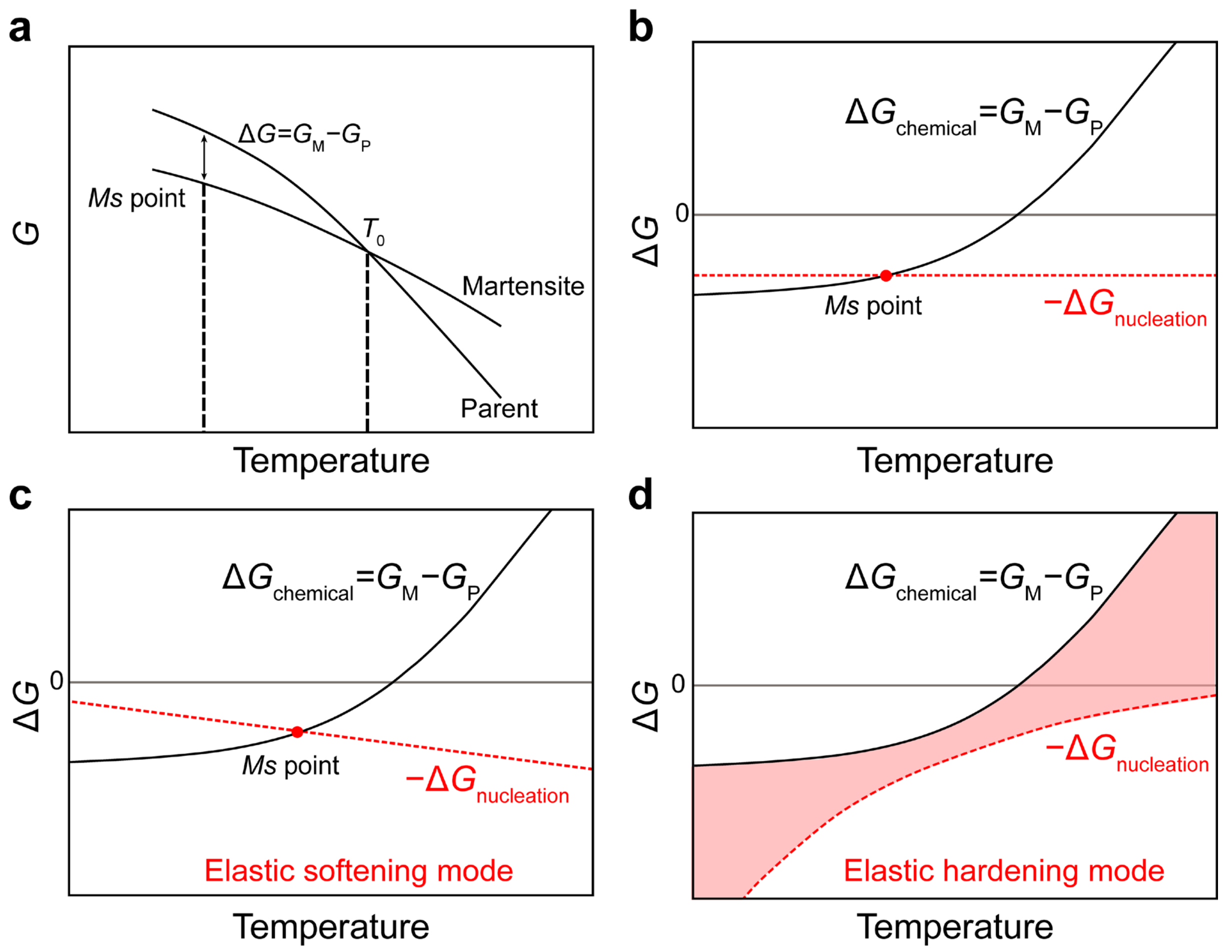

Supplementary Fig. 15 Schematic representation on the martensitic transformation. a, Schematic representation of the chemical Gibbs free energy curves for both parent and martensitic phases. **b-d,** Schematic representation of the chemical Gibbs free energy difference (solid line) and the driving force required for martensite nucleation (red dashed line) as a function of temperature, with the intersection point indicating the *Ms* point (the temperature at which martensitic transformation starts). **b,** Without considering temperature-dependent lattice instability or elastic modulus, the driving force required for martensite nucleation remains constant. **c,** In most shape-memory alloys showing elastic or phonon softening behavior, the driving force for martensite nucleation decreases with decreasing temperature, increasing the *Ms* point. **d,** In cases involving elastic hardening, such as the case of Ti-Al-Cr alloy, the driving force required for martensite nucleation increases with decreasing temperature, while the chemical Gibbs free energy difference increases more slowly during cooling. As a result, no intersection occurs, indicating the absence of thermally induced martensitic transformation.

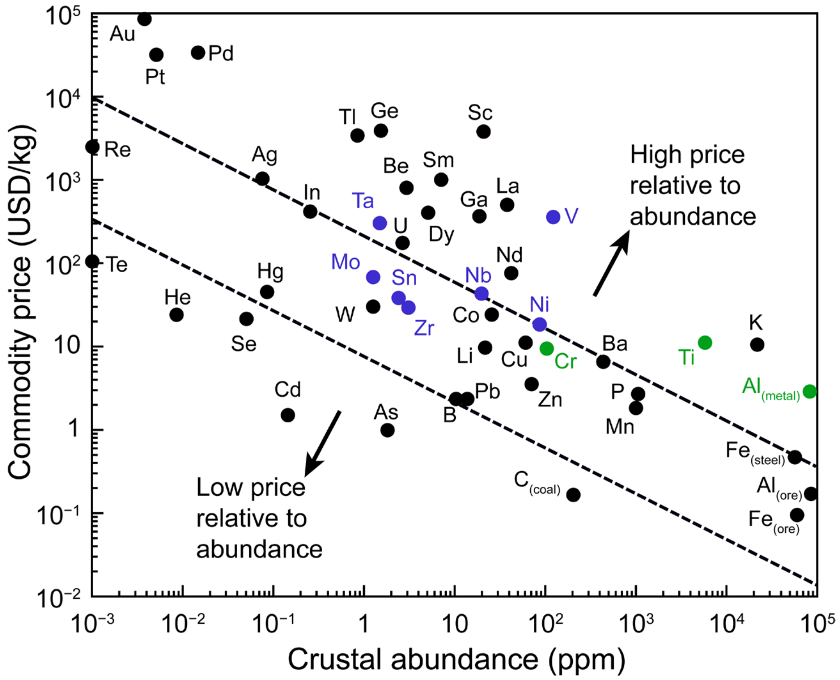

Supplementary Fig. 16 Price versus crustal abundance for some selected elements [11, 12].

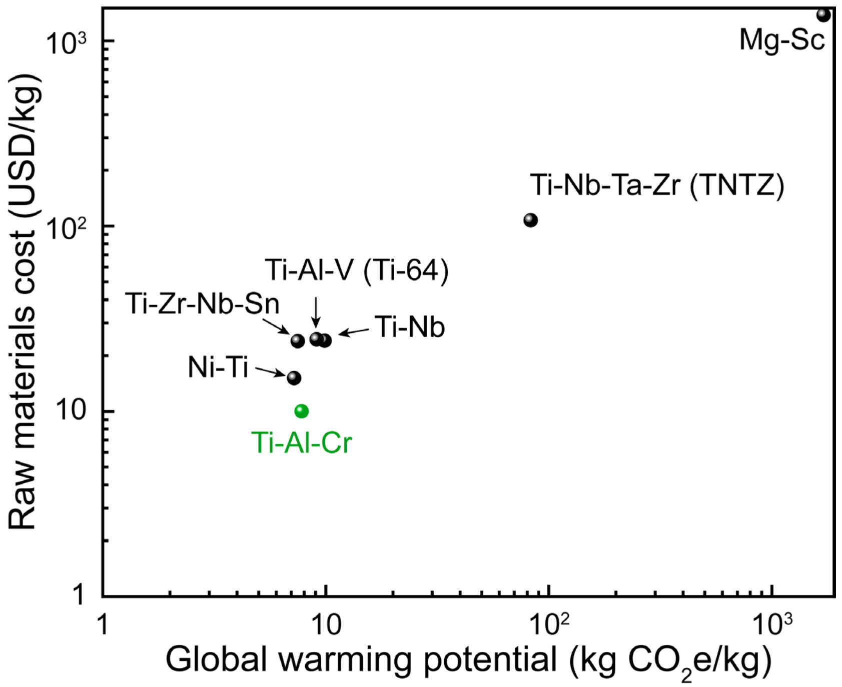

Supplementary Fig. 17 Raw materials cost versus global warming potential for the production of selected materials [13, 14]. Here, global warming potential refers to the equivalent mass of carbon dioxide emissions during the production of 1 kg of metals, serving as an indicator for evaluating the environmental burden.

Supplementary Table 1 Elastic constants for the present Ti-Al-Cr alloy experimentally determined at room temperature and some related elastic properties.

C_{11} (GPa)	C_{12} (GPa)	C_{44} (GPa)	C' (GPa)	B (GPa)	A
129.5	101.8	62.8	13.9	111.0	4.5

A: Index of elastic anisotropy, $A = C_{44}/C'$.

Supplementary Video 1 In situ optical surface observation during the tensile testing of a Ti-Al-Cr dog-bone shaped specimen at room temperature.

Supplementary References

1. Gopal, E. S. R. *Specific Heats at Low Temperatures* (Plenum, 1966).
2. Somsen, C., Wassermann, E. F., Kästner, J. & Schryvers, D. Precursor phenomena in a quenched and aged $Ni_{52}Ti_{48}$ shape memory alloy. *J. Phys. IV* **112**, 777 (2003).
3. Chernenko, V. A., Fujita, A., Besseghini, S. & Pérez-Landazabal, J. I. Low-temperature specific heat of Ni–Mn–Ga ferromagnetic shape memory alloys. *J. Magn. Magn. Mater.* **320**, 156-159 (2008).
4. Otsuka, K. & Wayman, C. M. *Shape Memory Materials* (Cambridge University Press, 1999).
5. Olson, G. B. & Cohen, M. Thermoelastic behavior in martensitic transformations. *Scr. Metall.* **9**, 1247-1254 (1975).
6. Olson, G. B. & Cohen, M. A general mechanism of martensitic nucleation: Part III. Kinetics of martensitic nucleation. *Metall. Trans. A* **7**, 1915-1923 (1976).
7. Clapp, P. C. A localized soft mode theory for martensitic transformations. *Phys. Status Solidi B* **57**, 561-569 (1973).
8. Clapp, P. C. Localized soft modes and ultrasonic effects in first order displacive transformations. *Mater. Sci. Eng.* **38**, 193-198 (1979).
9. Nakanishi, N. Elastic constants as they relate to lattice properties and martensite formation. *Prog. Mater. Sci.* **24**, 143-265 (1980).
10. Ohnuma, I., Fujita, Y., Mitsui, H., Ishikawa, K., Kainuma, R. & Ishida, K. Phase equilibria in the Ti-Al binary system. *Acta Mater.* **48**, 3113-3123 (2000).
11. Data collected from https://en.wikipedia.org/wiki/Prices_of_chemical_elements on September 26, 2024.
12. Data collected from https://www.metal.com/ on September 26, 2024.
13. Nuss, P. & Eckelman, M. J. Life cycle assessment of metals: a scientific synthesis. *PloS One* **9** e101298 (2014).
14. Raabe, D. The materials science behind sustainable metals and alloys. *Chem. Rev.* **123**, 2436-2608 (2023).
